# The Role of Cold-Sensitive Ion Channels in Peripheral Thermosensation

**DOI:** 10.3389/fncel.2020.00262

**Published:** 2020-08-20

**Authors:** Tamara Joëlle Buijs, Peter Anthony McNaughton

**Affiliations:** Wolfson Centre for Age-Related Diseases, King’s College London, London, United Kingdom

**Keywords:** cold sensation, TRPM8, TRPA1, TRPC5, ENaC, K2P, GluK2, CNGA3

## Abstract

The detection of ambient cold is critical for mammals, who use this information to avoid tissue damage by cold and to maintain stable body temperature. The transduction of information about the environmental cold is mediated by cold-sensitive ion channels expressed in peripheral sensory nerve endings in the skin. Most transduction mechanisms for detecting temperature changes identified to date depend on transient receptor potential (TRP) ion channels. Mild cooling is detected by the menthol-sensitive TRPM8 ion channel, but how painful cold is detected remains unclear. The TRPA1 ion channel, which is activated by cold in expression systems, seemed to provide an answer to this question, but whether TRPA1 is activated by cold in neurons and contributes to the sensation of cold pain continues to be a matter of debate. Recent advances have been made in this area of investigation with the identification of several potential cold-sensitive ion channels in thermosensory neurons, including two-pore domain potassium channels (K2P), GluK2 glutamate receptors, and CNGA3 cyclic nucleotide-gated ion channels. This mini-review gives a brief overview of the way by which ion channels contribute to cold sensation, discusses the controversy around the cold-sensitivity of TRPA1, and provides an assessment of some recently-proposed novel cold-transduction mechanisms. Evidence for another unidentified cold-transduction mechanism is also presented.

## Introduction

All biological processes are affected by temperature, so to maintain optimal function in the face of external thermal challenges it is crucial for animals to detect the temperature both of their bodies and the environment, and to react appropriately. The somatosensory neurons that sense external temperature are pseudo-unipolar cells whose cell bodies are located in the dorsal root ganglia (DRG), located alongside the spinal cord. Activation of thermal transduction mechanisms in the sensory nerve endings of these neurons leads to depolarization and consequently firing of action potentials along the axons that carry information about the intensity and duration of the stimulus to the spinal cord. This information is then relayed to the brain *via* the spinothalamic tract (Palkar et al., 2015).

**Figure 1:**
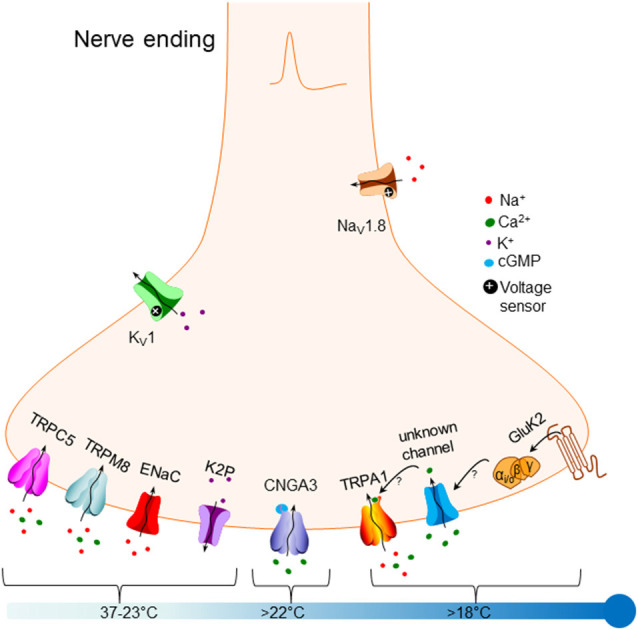
Overview of molecular mechanisms that have been proposed to underlie responses to cold in primary somatosensory neurons. Bottom left: potential sensory mechanisms triggered by mild non-noxious cooling, down to around 23°C, include TRPC5 [‘Transient Receptor Potential Canonical 5 (TRPC5) ” section], TRPM8 [“Transient Receptor Potential Melastatin 8 (TRPM8)” section], ENaC channels [“Epithelial Sodium Channels (ENaC)” section] and members of the K2P two-pore potassium channel family [“Two-Pore Domain Potassium Channels (K2P)” section]. Bottom middle: the cyclic nucleotide-gated channel CNGA3 has been proposed to be activated by cold down to 22°C [“Cyclic Nucleotide-Gated Channel Alpha 3 (CNGA3)” section]. Bottom right: noxious cold below 22°C has been proposed to activate TRPA1 [“Transient Receptor Potential Ankyrin 1 (TRPA1)” section], possibly *via* activation of an unknown channel (see arrows]. A recent article proposes that the glutamate receptor GluK2 [“Glutamate Ionotropic Receptor Kainate Type Subunit 2 (GluK2)” section] may trigger cold responses that are G-protein mediated, possibly by modulating an unknown channel (see arrows). Top right: expression of the Nav1.8 sodium channel may allow the cold sensation to continue down to very low temperatures (“Modulation of Cold Responses by Background Potassium Currents” section). Top left: sensory neuronal excitability can be modulated by the activity of members of the Kv1 potassium channel family, which are not directly cold-sensitive themselves (“Mechanisms Transmitting Cold Responses” section).

Cold activation of peripheral nerves can produce one of two sensations: moderate innocuous cold produces a sensation of pleasant coolness, while noxious cold produces a sensation of pain that triggers reflexes allowing animals to avoid tissue damage. Information about the cold is conducted by thinly myelinated Aδ-fibers and unmyelinated C-fibers. There are two types of cold-sensitive C-fiber: low-threshold fibers that are activated around 28°C and fire action potentials at a high rate, producing a sensation of coolness; and high-threshold fibers, also typically heat- and mechano-sensitive, that are activated around 5°C and fire action potentials at a slow rate, producing a sensation of pain (Grossmann et al., 2009). Several recent reviews have covered aspects of the molecular basis of cold sensation in mammals (Himmel and Cox, 2017; Lamas et al., 2019; MacDonald et al., 2020). In this review, we focus on the detection of external cold temperature mediated by peripheral somatosensory nerve fibers. Figure 1 outlines the main mechanisms that have been proposed to date.

## Cold-Sensitive TRP Channels

Most sensory transduction is mediated by ion channels. For example, mechanosensation is mediated by Piezo ion channels (Coste et al., 2010) and sour taste is sensed by acid-sensing ion channels (ASICs; Lingueglia, 2007). The main sensors of temperature in the nervous system are ion channels of the transient receptor potential (TRP) family. TRP channels have six transmembrane domains and are permeable to cations, such as Na^+^, K^+^, and Ca^2+^ (Julius, 2013). When thermosensitive TRP channels are activated by temperature, they open to allow cations into the cell, which depolarizes the membrane, leads to the generation of action potentials that signal the sensation to the CNS, and also causes a rise in the intracellular calcium concentration, either by direct Ca^2+^ influx through the calcium-permeable TRP channels themselves, or by triggering activation of voltage-gated Ca^2+^ channels (Palkar et al., 2015).

The temperature-sensitivity of ion channels can be determined by measuring their Q_10_ value, which is defined as the change in current passing through the ion channel resulting from a 10°C change in temperature. Most ion channels have a Q_10_ value between 1 and 3, while thermo-sensitive ion channels are defined as those having a Q_10_ value greater than three. According to this definition, the cold-sensitive TRP channels are TRPM8, TRPA1, and TRPC5 (Wang and Siemens, 2015).

### Transient Receptor Potential Melastatin 8 (TRPM8)

TRPM8 is a non-selective cationic channel that can be activated by cold between 25°C and 18°C, with a Q_10_ value of 24 (McKemy et al., 2002; Peier et al., 2002; Brauchi et al., 2004). TRPM8 may be gated directly by cold, but is also weakly voltage-gated and cold could activate the channel by shifting its voltage-dependent activation curve in a positive direction relative to the level of the resting membrane potential (McKemy et al., 2002; Brauchi et al., 2004; Voets et al., 2004; Karashima et al., 2007; Zakharian et al., 2010). Direct activation of TRPM8 by cold in neurons was confirmed *via* overexpression in mouse hippocampal neurons, which renders them cold-sensitive (de la Peña et al., 2005). The TRPM family of ion channels also contains the warmth-sensitive TRPM2 ion channel, which is closely related to TRPM8, but other members of this family are thermally-insensitive (Togashi et al., 2006; Tan and McNaughton, 2016).

Consistent with its range of thermal activation *in vitro*, TRPM8 also plays an important role in the sensation of non-noxious cool temperatures *in vivo*. For example, TRPM8 deficient mice have attenuated responses to the evaporative cooling assay, However, these mice can still sense noxious cold (Bautista et al., 2007; Dhaka et al., 2007). Even TRPM8/TRPA1 double KO mice display no deficits in noxious cold sensation (Brenner et al., 2014). Other studies, on the other hand, have reported that TRPM8 is required for both neural and behavioral responses to noxious cold in mice (Knowlton et al., 2010, 2011). On balance, TRPM8 plays a clear role in the sensation of non-noxious cool temperatures but is not the only cold transduction mechanism. Which other candidates have been proposed?

### Transient Receptor Potential Canonical 5 (TRPC5)

TRPC5 is a nonselective cationic channel that is activated in expression systems by a fall in temperature in the range between 25–37°C, with a Q_10_ value of ~10 (Okada et al., 1998; Zimmermann et al., 2011). The channel was found to contribute to cold responses of DRG neurons *in vitro*, but TRPC5 KO mice displayed no difference in temperature preference compared to WT mice (Zimmermann et al., 2011), suggesting that the ion channel is not involved to any significant extent in physiological cold-sensation.

### Transient Receptor Potential Ankyrin 1 (TRPA1)

TRPA1 is a nonselective cationic channel that can be activated by cold below 17°C in some expression systems, with a Q_10_ value of ~10 (Story et al., 2003; Sawada et al., 2007; Karashima et al., 2009). TRPA1 can be activated by various other noxious stimuli, including toxic bacterial products and environmental irritants (Viana, 2016). hTRPA1 inserted in lipid bilayers is activated by both cold and heat, conferring a U-shaped thermal sensitivity (Moparthi et al., 2014, 2016). However, recombinant rat and human TRPA1 when overexpressed in human embryonic kidney (HEK293) cells failed to respond to a 5°C cold stimulus, suggesting that the cold-sensitivity of TRPA1 was not intrinsic but resulted from a variable factor present in some cells but not others (Jordt et al., 2004; Nagata et al., 2005). hTRPA1 can be directly activated by [Ca^2+^]_i_ (Doerner et al., 2007; Zurborg et al., 2007; Moparthi et al., 2020), so some of these contradictory findings could be explained by indirect activation of TRPA1 *via* a background Ca^2+^ influx caused by cooling. Such a background cold-activated [Ca^2+^]_i_ increase has been observed in most cell lines (Caspani and Heppenstall, 2009).

Experiments addressing the cold-sensitivity of TRPA1 in neuronal cell cultures also remain inconclusive. One study found that TRPA1 was expressed by 46.5% of rat DRG neurons, but only 3.6% of DRG neurons responded to a noxious cold stimulus (Jordt et al., 2004). Similarly, 96% of TRPA1-expressing rat trigeminal ganglion (TG) neurons did not respond to a 5°C cold stimulus, as measured by *in vitro* Ca^2+^ imaging (Jordt et al., 2004). These experiments indicate that the expression of TRPA1 alone is not sufficient to generate cold responses. Additionally, neurons of TRPM8 KO mice that remained cold-sensitive in the absence of TRPM8, did not respond to the TRPA1 agonist mustard oil, showing that TRPA1 does not underlie their cold responses (Bautista et al., 2007). Only a small percentage of cold-sensitive mouse trigeminal neurons were activated by TRPA1 agonists (Madrid et al., 2009). In another study, only 8% of TRPA1-expressing DRG neurons responded to a 4°C cold stimulus in mice, showing that TRPA1 expression alone does not render neurons cold-sensitive, but the amplitude of the cold-activated Ca^2+^ influx in these neurons was reduced after application of the selective TRPA1 antagonist HC030031, suggesting that TRPA1 does contribute to their cold responses (Memon et al., 2017). Unfortunately, the authors did not provide data regarding the size of this decrease, which makes it difficult to conclude that TRPA1 was solely responsible for the observed Ca^2+^ influx. Therefore, these results do not prove that TRPA1 is directly cold-sensitive and the results could still be explained by indirect activation of TRPA1 *via* a background cold-activated Ca^2+^ influx, as suggested previously (Caspani and Heppenstall, 2009).

Behavioral studies using TRPA1 KO mice also report conflicting results. TRPA1 KO mice were indistinguishable from WT littermates in the two-plate thermal choice test (Bautista et al., 2006, 2007). Others have reported that TRPA1 did contribute to cold-sensation, but only in female mice (Kwan et al., 2006), or only in male mice (Winter et al., 2017). Another study found no significant difference between the responses of both male and female TRPA1/TRPM8 double KO mice when compared to their TRPM8 KO littermates (Brenner et al., 2014).

Others report that TRPA1 KO mice did exhibit a partial deficit in cold thermo-sensation, but only during some cold stimulus protocols (Karashima et al., 2009). TRPA1 may contribute, not to cold-sensation, but to cold hypersensitivity (del Camino et al., 2010; Tsagareli et al., 2019).it may not be required for any neural or behavioral responses to cold (Knowlton et al., 2010). An *in vivo* Ca^2+^ imaging study of the DRG showed no difference in responses to mild or intense cooling between TRPA1 KO and WT mice (Ran et al., 2016). In rats, the TRPA1 agonist cinnamaldehyde applied to the skin did not sensitize noxious cold-evoked hind limb withdrawal but did sensitize noxious *heat* withdrawal mediated by C-fibers (Dunham et al., 2010). In agreement, TRPA1 in mammalian somatosensory neurons has recently been found to be heat-sensitive in at least some neurons (Vandewauw et al., 2018), echoing its heat-activation in the exquisitely heat-sensitive thermal sensors of pit vipers (Gracheva et al., 2010; Chen et al., 2013). Furthermore, TRPA1 mediates itch-related heat hyperalgesia in mice (Tsagareli et al., 2019). Taken together, these studies indicate that TRPA1 is not a key contributor to physiological cold-sensation.

## Evidence for Alternative Cold Transduction Mechanisms

There are two populations of cold-sensitive neurons in the rat DRG responding to decreases of temperature between 32–12°C. One population (>70% of cold-sensitive neurons) is sensitive to the TRPM8 agonist menthol and therefore presumably expresses TRPM8, and a second population is not sensitive to menthol (Babes et al., 2004). Furthermore, knockout of TRPM8 in mice caused only a partial reduction in the number of DRG neurons that responded to cooling (14.9% to 7.6%; Dhaka et al., 2007). Therefore, another cold-sensitive mechanism must be present in mouse somatosensory neurons. These observations cannot be explained by the presence of TRPA1, because a third of cold-sensitive mouse DRG neurons lack both TRPM8 and TRPA1 channels (Munns et al., 2007).

The sympathetic nervous system is also intrinsically cold-sensitive. Postganglionic sympathetic neurons of the mouse superior cervical ganglion (SCG) can be directly activated by cold <16°C (Smith et al., 2004; Munns et al., 2007). A physiological function for this sensory mechanism has not yet been identified. Sympathetic nerves trigger cold defense mechanisms, such as cutaneous vasoconstriction, shivering thermogenesis, and brown adipose tissue thermogenesis (Morrison, 2016). Perhaps the thermo-sensitive properties of sympathetic neurons serve to enhance these functions. Around 60% of mouse SCG neurons express a cold-sensitive ion channel whose activation results in an influx of Ca^2+^. The Ca^2+^ influx is not activated by the TRP channels agonists menthol or mustard oil and so cannot be either TRPM8 or TRPA1 (Munns et al., 2007). Similarly, the cold-sensitivity of TG neurons that innervate the dental pulp is not mediated by either TRPM8 or TRPA1 (Michot et al., 2018). These studies suggest that there are at least two mechanisms of cold transduction: TRPM8, and an unknown Ca^2+^ influx mechanism that is not TRPA1.

Apart from TRP channels, a few other types of ion channel have been proposed to function as cold sensors, including two-pore domain potassium channels (K2P; Maingret et al., 2000; Kang et al., 2005), epithelial sodium channels (ENaC; Askwith et al., 2001), GluK2 glutamate receptors (Gong et al., 2019), and CNGA3 cyclic nucleotide-gated ion channels (Feketa et al., 2020). The possible contribution of these ion channels to cold sensation will now be discussed. A comparison of the thermosensitive properties of these ion channels is provided in Supplementary Table S1.

### Epithelial Sodium Channels (ENaC)

The constitutively active Na^+^ current of human cationic epithelial sodium channels (ENaC) can be potentiated by cold lower than 23–25°C with a Q_10_ value of 4.4 in heterologous cells and mouse DRG neurons, but only in the presence of protons (Askwith et al., 2001). Therefore, these channels could potentially play a role in cold transduction by depolarizing the membrane and triggering action potential firing. However, this hypothesis is challenged by the finding that the ENaC antagonist amiloride did not affect cold-activated currents in rat DRG neurons (Reid and Flonta, 2001).

### Two-Pore Domain Potassium Channels (K2P)

The K2P family consists of 15 genes that together make an important contribution to the native K^+^ background leak currents observed in many neurons. The main function of K2P channels is to control membrane excitability by setting the resting membrane potential (Enyedi and Czirják, 2010). These channels may form a cold transduction mechanism as follows: K2P channels are normally open at physiological temperatures but some close when exposed to cold, thus causing net depolarization of the cell; the membrane potential may thus reach the threshold, opening Na_V_ and Ca_V_ channels, and generating action potentials.

Of the K2P channels, mTREK1, rTREK1, rTREK2, hTREK2, rTRAAK, mTASK3, hTASK3, and mTRESK are thermosensitive (Lesage et al., 2000; Maingret et al., 2000; Kang et al., 2005; Morenilla-Palao et al., 2014; Castellanos et al., 2020). *in vitro*, the deletion of TREK1 or TRAAK alone does not affect cold-sensitivity, but a double KO increases the percentage of cold-sensitive mouse DRG neurons from 24% to 54%. Notably, this mechanism mostly affects neurons that do not express TRPM8 or TRPA1 (Noël et al., 2009). In contrast, TASK3 is enriched in a subpopulation of mouse TRPM8-expressing neurons where it shifts the cold-activation threshold from 29.9°C to 28°C (Morenilla-Palao et al., 2014).

KO mice have been generated to establish the function of these channels *in vivo*. TREK1 and TRAAK KO mice have no obvious phenotype (Heurteaux et al., 2004; Alloui et al., 2006; Noël et al., 2009), but TREK1/TRAAK double KO mice are more sensitive to cold in the cold plate assay and temperature preference test (Noël et al., 2009). Similarly, both TREK2 and TRESK KO mice have a somewhat enhanced sensitivity to moderately cool temperatures (Pereira et al., 2014; Guo et al., 2019; Castellanos et al., 2020). The studies summarized above suggest a role for K2P channels in thermosensation. Whether the inhibition of the background K^+^ current mediated by these channels solely *modulates* neuronal excitability or constitutes a cold-transduction mechanism in isolation, needs further study.

### Glutamate Ionotropic Receptor Kainate Type Subunit 2 (GluK2)

A recent genetic screen has put forward another candidate cold sensor by showing that the glutamate receptor GLR-3 is necessary for cold-avoidance behavior in Caenorhabditis elegans (Gong et al., 2019). GLR-3 is both an ionotropic and metabotropic receptor, coupled to the inhibitory protein G_i/o_. The mammalian homolog GluK2 also responds to cold <18°C when overexpressed in Chinese Hamster Ovary (CHO) cells as measured by Ca^2+^ imaging, and this Ca^2+^-influx is reduced in mouse DRG neurons treated with mGluk2 siRNA *in vitro*. Curiously, the observed cold-activated Ca^2+^-influx was shown to be independent of the ionotropic function of GluK2 itself. Furthermore, it was abolished in the absence of extracellular Ca^2+^, indicating that this Ca^2+^ increase is not mediated by Ca^2+^-release from intracellular stores. Therefore, it must be mediated by another unidentified membrane channel present in all cell types tested, including CHO, COS-7, Hela, and DRG neurons (Gong et al., 2019). The molecular pathway by which the activation of an inhibitory G_i/o_-coupled receptor such as Gluk2 can lead to activation of a Ca^2+^ channel needs further study.

### Cyclic Nucleotide-Gated Channel Alpha 3 (CNGA3)

Another novel candidate for the unidentified cold sensor is the cation channel CNGA3. CNGA3 is not directly gated by cold, but its activation by cGMP can by potentiated by cold below 22°C, with a Q_10_ value of 6.5 (Feketa et al., 2020). The cation channel CNGA3 was first discovered to be cold-sensitive in Grünenberg ganglion neurons, located in the vestibule of the murine nose (Stebe et al., 2014). These neurons transduce coolness *via* a cGMP cascade (Mamasuew et al., 2010). Additionally, CNGA3 is responsible for cold responses of a subpopulation of neurons in the thermoregulatory center of the hypothalamus in mice (Feketa et al., 2020). Interestingly, CNGA3 is also enriched in a subpopulation of cold-sensitive DRG neurons (Luiz et al., 2019). Further research is needed to determine whether CNGA3 contributes to cold responses in these neurons.

### Modulation of Cold Responses by Background Potassium Currents

The temperature threshold of cold-sensitive neurons is determined not only by the expression of cold transduction channels but can also be modulated by a cold-insensitive “excitability brake current,” *I*_KD_, carried by K^+^ ions, which controls neuronal excitability (Viana et al., 2002). *I*_KD_ has been reported to play a role in cold-sensitive mouse neurons expressing either TRPM8 or TRPA1 (Madrid et al., 2009; Memon et al., 2017). It is thought to be mediated by K_V_1 channels, as it can be blocked by dendrotoxins (Madrid et al., 2009; Teichert et al., 2014). When activated, *I*_KD_ causes hyperpolarisation of the sensory nerve, making it less sensitive to depolarization by cold-sensitive ion channels.

### Mechanisms Transmitting Cold Responses

Transmission of the sensation of extreme cold to the CNS has been the subject of two interesting studies. Zimmermann et al. (2007) found that while many mechanisms mediating exonal excitability were inhibited by strong cold, activation of the sodium channel Na_V_1.8 was maintained, with the result that conduction of action potentials, and hence the sensation of noxious cold, was maintained in normal mice but was lost in mice in which Na_V_1.8 had been deleted (Zimmermann et al., 2007; Abrahamsen et al., 2008). A more recent article found, in contrast, that Na_V_1.8 was in general not expressed in the same neurons as the cold sensor TRPM8, nor in neurons responding to cold down to 5°C. Moreover, the deletion of Na_V_1.8 had little effect on cold responses until the temperature had reached strongly noxious levels (<5°C; Luiz et al., 2019). Both articles agree, though, that responses to extreme cold (<5°C) are ablated by the deletion of Na_V_1.8.

## Concluding Remarks

Cold is sensed by specialized sensory nerve endings in the periphery. In these nerve endings, a combination of ion channels is responsible for transducing the sensation of cold. The role of TRPM8 in *innocuous* cold sensation has been well established, but which combination of cold transduction molecules is responsible for the sensation of *noxious* cold remains unclear. The vexed question of whether TRPA1 accounts for any fraction of noxious cold sensation has entertained the field for the last decade or more. It is clear at least that TRPA1 is not the only noxious cold sensory mechanism, because: (a) cold-sensitive neurons remaining after deletion of TRPM8 do not in general express TRPA1; (b) most TRPA1-expressing neurons are not cold-sensitive; and (c) mice still exhibit strong cold-aversive responses after deletion of both TRPM8 and TRPA1. On balance, it seems clear that any contribution of TRPA1 to noxious cold sensation is small, and other mechanisms must exist.

Several studies provide evidence for the presence of an unidentified Ca^2+^ influx mechanism activated by noxious cold *in vitro* (Smith et al., 2004; Munns et al., 2007; Gong et al., 2019). Novel candidates for this mechanism have been proposed recently, none of which belong to the TRP ion channel family. Each of these discoveries raises further questions: (a) K2P channels make a major contribution to the neuronal resting potential, and action potentials can be initiated when the activity of these channels is suppressed by cold. Do K2P channels form an independent cold transduction mechanism, or do these channels function exclusively to *modulate* cold responses? (b) GluK2, which appears to act both as an ionotropic glutamate receptor and as a metabotropic cold sensor, has recently been suggested as a novel cold-sensitive mechanism. But how can activation of an *inhibitory* G protein-coupled receptor such as GluK2 lead to action potential firing? and (c) The cGMP-gated receptor CNGA3 has also recently emerged as a candidate cold sensor in the vomeronasal organ of rodents. By which pathway might CNGA3 mediate cold transduction in somatosensory nerves? Answering these questions is crucial for completing our understanding of the mechanisms that underlie cold sensation. Future research may provide a much-needed target for pharmaceutical intervention in patients that suffer from hypersensitivity to cold, such as those with fibromyalgia, chemotherapy-induced cold hypersensitivity, and dental cold hypersensitivity.

## Author Contributions

TB and PM wrote the manuscript.

## Conflict of Interest

The authors declare that the research was conducted in the absence of any commercial or financial relationships that could be construed as a potential conflict of interest.
