# Supplementary material for: The Role of Cold-Sensitive Ion Channels in Peripheral Thermosensation
Source: Front Cell Neurosci. 2020 Aug 20;14:262. doi: 10.3389/fncel.2020.00262 (PMC7468449; doi:10.3389/fncel.2020.00262)
Supplement: Supplementary file 1 [file Table_1.DOCX]

Table 1 A comparison of the thermosensitive properties of all candidate cold sensors proposed to date. This table also provides references to in vitro and in vivo studies that examined the function of these ion channels in neuronal cold sensation.

| **Type** | **Channel** | **Threshold** | **Q_10_** | **Contributes to neuronal cold sensation *in vitro*?** | **Contributes to neuronal cold sensation *in vivo?*** |
| --- | --- | --- | --- | --- | --- |
| TRP | TRPM8 | <28˚C | 24 | Yes, in DRG (Dhaka et al., 2007). | Yes (Bautista et al., 2007; Colburn et al., 2007) |
|  | TRPC5 | 37˚C-25˚C | 10 | Yes, in DRG (Zimmermann et al., 2011). | No (Zimmermann et al., 2011) |
|  | TRPA1 | <17˚C | 10 | Yes, in DRG (Karashima et al., 2009; Memon et al., 2017)  Not in DRG (Bautista et al., 2007).  Not in TG (Jordt et al., 2004; Madrid et al., 2009) | No (Bautista et al., 2006, 2007; Dunham et al., 2010; Knowlton et al., 2010; Ran et al., 2016)  Only in some tests (Kwan et al., 2006; Karashima et al., 2009; del Camino et al., 2010; Brenner et al., 2014; Winter et al., 2017) |
| ENaC | ENaC | 25˚C-23˚C | 4.4 | Yes, in DRG (Askwith et al., 2001). | N/A |
| K2P | TREK1 | 32˚C-37˚C | 7 | Only in combination with TRAAK in DRG (Noël et al., 2009). | Only in combination with TRAAK (Heurteaux et al., 2004; Alloui et al., 2006; Noël et al., 2009). |
|  | TREK-2 | >25˚C | 14 | Yes, in DRG (Pereira et al., 2014). | Yes (Pereira et al., 2014). |
|  | TRAAK | >31˚C | N/A | Only in combination with TREK-1 in DRG (Noël et al., 2009). | Only in combination with TREK-1 (Heurteaux et al., 2004; Alloui et al., 2006; Noël et al., 2009). |
|  | TASK-3 | 20˚C-33˚C | N/A | Only in some TRPM8-expressing DRG neurons (Morenilla-Palao et al., 2014). | Yes (Morenilla-Palao et al., 2014). |
|  | TRESK | >25˚C | N/A | Yes, in DRG (Castellanos et al., 2020). | No (Guo et al., 2019).  Yes (Castellanos et al., 2020). |
| Glu | GluK2 | <18˚C | N/A | Yes, in DRG (Gong et al., 2019). | N/A |
| CNG | CNGA3 | <22 ˚C | 6.5 | Yes, in Grünenberg ganglion (Mamasuew et al., 2010)  Yes, in hypothalamus (Feketa et al., 2020). | N/A |
